# DOES PERSONALITY PREDICT PATTERNS OF AGING? LONGITUDINAL FINDINGS FROM THE VA NORMATIVE AGING STUDY

**DOI:** 10.1093/geroni/igz038.2655

**Published:** 2019-11-08

**Authors:** Ritwik Nath, Carolyn M Aldwin, Soyoung Choun, Maria Kurth, Avron Spiro III

**Affiliations:** 1 OSU Oregon State University, Corvallis, Oregon, United States; 2 Boston University, Boston, Massachusetts, United States

## Abstract

Previous research using the Veterans Affairs Normative Aging Study identified four patterns of aging using group-based multi-trajectory modelling to identify joint changes in life satisfaction (LS) and functional health (FH) (Nath et al., 2018a,b). The purpose of the present study was to examine whether personality traits predicted these four patterns: impaired (stable low LS and FH), normal (decreasing LS and FH), optimal (high LS, decreasing FH), and successful aging (high LS and FH). The sample consisted of 992 NAS men who provided 3,853 observations (M=2.81, SD=1.54, range 1–8) between 1987 and 2010 (Mage=62.31, SDage=7.50, range 44-86 in 1987). Multinomial logit regression analysis with robust estimation controlled for marital, employment, and self-rated health status, using optimal aging as the reference group. Compared to the optimal aging group, neuroticism predicted membership to the normal aging (RR=1.61, CI=1.16–2.22) and the successful aging groups (RR=.55, CI=.38–.79). Optimism predicted membership to all groups, with lower risk ratios to the impaired aging group (RR=.11, CI=.04–.33) and normal aging group (RR=.57, CI=.42–.79), and higher risk ratio to the optimal aging group (RR=1.96, CI=1.35–2.85). Extraversion only predicted membership in the impaired aging group (RR=.27, CI=.12–.63) indicating high levels of social isolation. Thus, personality may play an important role in determining the patterns of aging.

